# Conservative finite-difference scheme for the problem of THz pulse interaction with multilevel layer covered by disordered structure based on the density matrix formalism and 1D Maxwell’s equation

**DOI:** 10.1371/journal.pone.0201572

**Published:** 2018-08-02

**Authors:** Vyacheslav A. Trofimov, Svetlana A. Varentsova, Irina G. Zakharova, Dmitry Yu. Zagursky

**Affiliations:** 1 Faculty of Computational Mathematics and Cybernetics, Lomonosov Moscow State University, Moscow, Russia; 2 Faculty of Physics, Lomonosov Moscow State University, Moscow, Russia; Universidad Miguel Hernandez de Elche, SPAIN

## Abstract

On the basis of the Crank-Nicolson method, we develop a conservative finite-difference scheme for investigation of the THz pulse interaction with a multilevel medium, covered by a disordered layered structure, in the framework of the Maxwell-Bloch equations, describing the substance evolution and the electromagnetic field evolution. For this set of the partial differential equations, the conservation laws are derived and proved. We generalize the Bloch invariant with respect to the multilevel medium. The approximation order of the developed finite-difference scheme is investigated and its conservatism property is also proved. To solve the difference equations, which are nonlinear with respect to the electric field strength, we propose an iteration method and its convergence is proved. To increase the computer simulation efficiency, we use the well-known solution of Maxwell’s equations in 1D case as artificial boundary condition. It is approximated using Cabaret scheme with the second order of an accuracy. On the basis of developed finite-difference scheme, we investigate the broadband THz pulse interaction with a medium covered by a disordered structure. This problem is of interest for the substance detection and identification. We show that the disordered structure dramatically induces an appearance of the substance false absorption frequencies. We demonstrate also that the spectrum for the transmitted and reflected pulses becomes broader due to the cascade mechanism of the high energy levels excitation of molecules. It leads to the substance emission at the frequencies, which are far from the frequency range for the incident pulse spectrum. Time-dependent spectral intensities at these frequencies are weakly disturbed by the disordered cover and, hence, they can be used for the substance identification.

## 1. Introduction

The detection and identification of hazardous chemical, biological and other substances by using the remote sensing is in research focus. One of the ways to achieve this aim is THz TDS because the THz radiation is non-ionizing and most common substances are transparent to it. Moreover, many hazardous substances have spectral fingerprints in the THz range of frequencies [1–10]. As a rule, the substance identification is based on a comparison of the absorption frequencies for a substance under investigation with the corresponding frequencies from a database. This method is used not only for security applications but also for a molecular structure investigation [11–15], nondestructive inspection [16–18], medical application [19], food quality inspection [20]. Currently, nanoscale terahertz spectroscopy is being actively developed as well [21].

Despite the obvious advantages of THz TDS using for the substance detection and identification, there is some shortcoming of its application [22]. Apparently, under real conditions, the factors like opaque packaging, inhomogeneity of the substance surface, atmospheric humidity and others affect the THz spectroscopy results [23–27]. In addition, a substance is usually covered by ordinary materials (paper, clothes or some packaging) [28]. Being made from materials transparent for a THz radiation these covers often possess a microscopic mechanical structure in the sub-millimeter scale of lengths. This leads to cover density fluctuations and also to its thickness variations. Therefore, the dielectric permittivity of such covers is not homogeneous and can be considered as a disordered photonic structure in the THz frequency range. Thus, observing a THz pulse reflected from a neutral material, we can reveal the false absorption frequencies, which are not inherent to this material, and the material may be wrong considered as dangerous one [29–32]. The overcoming of this serious drawback of the THz TDS method mentioned above and its physical mechanism understanding are the challenging problems for developing the real detection systems.

Another significant feature of the pulsed THz TDS is the THz pulse broadening after its interaction with a substance. Usually, the spectrum of the THz pulse transmitted through or reflected from a medium contains frequencies, which are, for example, greater than the maximal frequency of the incident pulse spectrum. This effect cannot be explained by multi-photon absorption of the THz radiation, because the THz pulse intensity is usually insufficient for its appearance. Using the computer simulation we have demonstrated a new spectral harmonic generation mechanism due to the absorption of a photon sequence (cascade mechanism), which results in the molecule higher energy levels excitation [32]. This mechanism may be observed in a great number of materials, possessing of vibration or rotation energy levels. We note that a photon sequence absorption belonging to visible frequency range was observed at studying the two-photon luminescence in a semiconductor [33].

It is well-known that the excited molecules relax to the equilibrium states. Therefore, the emission frequencies in the reflected or transmitted signal spectrum appear. The substance emission at high frequencies can be used for the substance identification because a radiation with a shorter wavelength is less affected by the cover material. The computer simulation is based on the Maxwell-Bloch equations [34], [35].

We emphasize that in our knowledge a problem statement under consideration is novel one and has not been investigated earlier. There are, at least, three features, which allow to state this. The first of them is an analysis of the interaction of the THz pulse containing a few cycles with disordered (or ordered) structure. The well-known Transfer Matrix Method for the description of the electromagnetic radiation interaction with the periodic structure is inapplicable in the case under consideration.

The second one is the following. An active medium (i.e. the medium capable of absorbing and emitting the THz waves), which is placed inside the disordered structure, can be considered as an impurity of the periodical structure. In optics, the sizes of such impurities are much less than the sizes of the photonic crystal elements. In our case, the active medium length is comparable with or greater than a length of the disordered structure layers.

The third of the mentioned features is the description of the active medium response to the THz pulse action. We describe its characteristics evolution in the framework of a matrix density formalism.

Usually, computer simulation of the electromagnetic field interaction with a substance describing in the framework of the Maxwell-Bloch equations is based on a combination of the FDTD method [36] with the step-split method [34], [37]. As we believe, for the first time, the Crank-Nicolson scheme for the time discretization of the Bloch equations was applied for a two-level medium in [38]. However, the Crank-Nicholson scheme does not possess the positiveness property of the density matrix diagonal elements for three and more energy levels. In [39] it was shown that negative values of the energy levels populations may even occur in numerical simulation. In the same paper [39] and later in [40] a step-split method was used for solution of the matrix density elements equations with respect to the multilevel medium. This approach guarantees the positiveness of the density matrix diagonal elements but it leads to the decoupling of the Maxwell-Bloch equations. The latter feature leads to non- preserving the integrals of motion (invariants).

Below we derive a conservation law, which is a generalization of the Bloch invariant with respect to the multilevel medium. For computer simulation, we use a finite-difference scheme based on the Crank-Nicolson scheme supplemented by artificial boundary conditions, which approximate the well-known solution of the 1D wave equation [41]. Using the Cabaret scheme [42], we achieve the second order of accuracy for its approximation. Essentially that the proposed finite-difference scheme also possesses the conservativeness property with respect to the derived Bloch invariant and the invariants of the Maxwell’s equations. To realize the finite-difference scheme we apply an iteration process and prove its convergence. Computer simulations show that the positiveness property of the density matrix diagonal elements is satisfied by choosing a mesh time step, which is defined by the problem parameters.

This article is organized in the following way. In the Section 2, we state the problem and derive its invariants.

In the Section 3, we develop the conservative finite-difference scheme for the Maxwell-Bloch equations formulated in the previous Section. Since the finite-difference scheme is a nonlinear one, we solve it by using an iterative method. We prove the theorem of the iteration convergence.

Finally, in the Section 4 we discuss the computer simulation results for the THz pulses transmitted through (or reflected from) an uncovered medium or a medium covered by disordered structure. Then we discuss the differences in spectra of these pulses and a physical mechanism, which causes such differences.

## 2. Problem statement

Initially, a THz pulse with a few cycles is located in vacuum and falls on an optically active medium (i.e. a medium, capable of electromagnetic field energy absorbing or emitting at specific frequencies). This medium is placed inside a disordered layered structure. Each of layers is characterized by its inherent dielectric permittivity and all layers are transparent for the THz radiation. The latter means that the layers do not possess any absorption frequencies in the THz frequency range. Below, the term “medium” will denote the active medium and the term “cover” or “covering” will denote a disordered layered structure.

**Fig 1:**
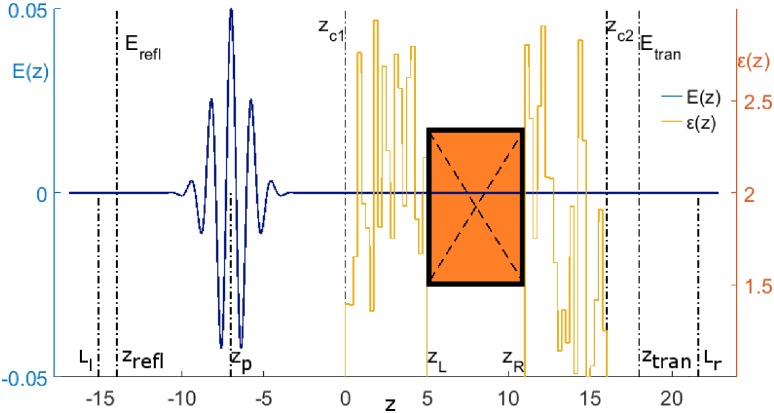
The scheme of the THz pulse interaction with covered substance. The left coordinate axis shows the electric field strength in dimensionless units. The dielectric permittivity of layers is shown on the right axis. The crossed rectangle depicts the active medium. Lines marked as *E*_*refl*_, *E*_*trans*_ denote coordinates at which the reflected and transmitted THz pulses are measured.

A scheme of the laser pulse propagation is shown in Fig 1. Several important coordinates are marked in the figure, namely: positions of electric field detectors *E*_*refl*_ and *E*_*trans*_; coordinates of the active substance faces *z*_*L*_ and *z*_*R*_, and coordinates of the cover faces *z*_*c1*_ and *z*_*c2*_. The pulse propagates in vacuum, then transmits through the left cover, a medium, and finally, through the right cover, and exits into vacuum to the right. The pulse is partly reflected from various boundaries between layers and from the medium faces too. A certain part of the pulse energy is absorbed by the active medium. Generally speaking, a part of the absorbed energy can be emitted by the medium due to radiative transitions (emission) between excited energy levels. Reflected and transmitted pulses are detected near the left and right boundaries of the computational domain at the sections *E*_*refl*_ and *E*_*trans*_. As a rule, these pulses may consist of a sequence of the THz sub-pulses.

### 2.1 Maxwell-Bloch equations

We use the Maxwell’s set of equations describing the THz pulse propagation in 1D case:

$$-\frac{1}{с}\frac{\partial B}{\partial t}=\frac{\partial E}{\partial z}\;(\text{CGS}),\;-\frac{\partial B}{\partial t}=\frac{\partial E}{\partial z}\;(\text{SI}),$$ (1)

$$-\frac{1}{с}\frac{\partial D}{\partial t}=\frac{\partial H}{\partial z}\;(\text{CGS}),\;-\frac{\partial D}{\partial t}=\frac{\partial H}{\partial z}\;(\text{SI}),$$ (2)

$$\begin{array}{c} D=E+4\pi P(\text{CGS}),\;D=\varepsilon_{0}E+P(\text{SI}),\\ B=\mu H(\text{CGS}),\;B=\mu_{0}\mu H(\text{SI}), \end{array}$$ (3)

$$0<t\leq L_{t},\;L_{l}<z\leq L_{r},$$ (4)

together with equations with respect to the matrix density elements (see below). For convenience, we write above the set of equations in both systems of physical units. Here *H*, *E* are the magnetic field strength and the electric field strength, correspondingly. *B*, *D* are the magnetic field and electric field induction strength, correspondingly, *z* and *t* are the spatial coordinate and time, *c* = 3·10^8^ m·s^-1^ is the light velocity in a vacuum. Parameters *ε*_*0*_ and *μ*_*0*_ in Eqs (1)–(3) are the permittivity of free space and the magnetic permeability of free space and their values are equal to *ε*_*0*_ *=* 8.85·10^−12^ F·m^-1^, and *μ*_*0*_ *=* 4*π*·10^−7^ H·m^-1^ = 4*π*·10^−7^ T·m A^-1^, correspondingly [43]. Below we consider the case of a non-magnetic medium, thus, in Eq (3) the magnetic permeability is equal to *μ =* 1. *P* describes the medium polarization, *L*_*t*_ is a time interval during which the electromagnetic field propagation is analyzed. Parameters *L*_*l*_ and *L*_*r*_ (see Fig 1) denote spatial coordinates of the domain beginning and its ending. The total length of the domain is equal to *L*_*z*_. = *L*_*r*_*—L*_*l*_. Coordinates *z*_*R*_ and *z*_*L*_ mark the positions of the medium boundaries, *L*_*s*_. = *z*_*R*_*—z*_*L*_ is used to denote its length. We consider a TE mode of the THz pulse propagation.

The THz pulses reflected from and transmitted through the medium with covering are computed for a number of random realizations for the dielectric permittivities and then the result is averaged. This procedure is usually applied in physical experiments also to suppress a random fluctuation influence on the measured THz pulse characteristics. Let us notice that, each of two covering structures contains *N*_*l*_ layers with the layer thickness *h*_*l*_ (see Fig 1). Dielectric permittivities at each of the layers we denote as *ε*_*j*_, *ε*_*j*_′, *j* = [1, *N*_*l*_], for left and right covering, correspondingly.

Obviously, in vacuum (z ∈ [*L*_*l*_, *z*_*c*1_] ∪ [*z*_*c*2_, *L*_*R*_]) the electric field induction is equal to the electric field strength: *D = E*. In the cover layers (z ∈ [*z*_*c*1_, *z*_*L*_] ∪ [*z*_*R*_, *z*_*c*2_]) the polarization is assumed to be proportional to the electric field strength and the Eq (3) is replaced by the following formula:

D(z,t)=ε(z)E(z,t), (5)

where the dielectric permittivity *ε*(*z*) for each of the layers varies randomly in the range *ε*_*l*_ = [*ε*_min_, *ε*_max_] and it is uniformly distributed.

To describe the medium polarization (z ∈ [*z*_*L*_, *z*_*R*_]) we use the matrix density formalism. In this case the medium is described by the following set of equations:

$$\frac{\partial \rho_{mn}}{\partial t}=-(\gamma_{mn}+i\omega_{mn})\rho_{mn}+\frac{i}{\hbar }E\sum_{q=1}^{N}\left(d_{mq}\rho_{qn}-\rho_{mq}d_{qn}\right),$$ (6)

$$\frac{\partial \rho_{mm}}{\partial t}=-\sum_{q=1}^{N}\left(W_{mq}\rho_{mm}-W_{qm}\rho_{qq}\right)+\frac{i}{\hbar }E\sum_{q=1}^{N}\left(d_{mq}\rho_{qm}-\rho_{mq}d_{qm}\right),$$ (7)

$$P=M\sum_{n=1}^{N}\sum_{m=1}^{N}\left(d_{mn}\rho_{nm}\right)+\chi E,\;z_{L}<z<z_{R},\;0<t\leq L_{t},\;n,m=\bar{1,N}.$$ (8)

Here, *ρ*_*mn*_ = *ρ*_*mn*_(*z*,*t*) is the element of the density matrix describing the state of a molecule, which possesses *N* energy levels, in a time moment *t* and at the medium section *z*. Diagonal elements of the matrix *ρ*_*mn*_ describe the number of molecules at the energy level *m*. These elements obey the condition $\sum_{m=1}^{N}\rho_{mm}=1$ and obviously they are non-negative. The off-diagonal elements *ρ*_*mn*_ are related to the medium polarization corresponding to a transition between *n* and *m* energy levels. *γ*_*mn*_, *ω*_*mn*_ and *d*_*mn*_ are the relaxation rate of non-diagonal elements, the transition frequency, and the dipole moment. They are associated with a transition from energy level *m* to *n*, correspondingly, *W*_*mn*_ is the population decay rate. Parameter *M* is the density of “active” molecules in the medium, *χ* corresponds to the non-resonant linear electric susceptibility of the substance, *ћ* is the reduced Plank constant.

It should be noted that the Eqs (6)–(8) describe both linear and nonlinear interaction of the electromagnetic field with the multilevel medium in the dipole approximation. The term $P=M\sum_{n=1}^{N}\sum_{m=1}^{N}\left(d_{mn}\rho_{nm}\right)$ in the expression for the medium polarization (8) corresponds to a resonant or near-resonant response of the medium to the THz pulse action and causes the electromagnetic field energy absorption and emission of the medium. The second term *χE* in the same equation describes a linear non-resonant polarization of the medium arising because of the pulse interaction with the medium which has transition frequencies distant from the pulse carrier frequency. This term is responsible for the Fresnel reflection from the medium boundaries and plays the key role in the formation of reflected pulses.

The initial condition for the electromagnetic field is as follows:

$$\begin{array}{c} E(z,t=0)=E_{0}\cdot \text{exp}\left(\frac{-{\left(z-z_{p}\right)}^{2}}{a_{z}^{2}}\right)\cdot \text{cos}(\frac{2\pi \nu_{p}}{c}\cdot (z-z_{p})),\\ H(z,t=0)=H_{0}\cdot \text{exp}\left(\frac{-{\left(z-z_{p}\right)}^{2}}{a_{z}^{2}}\right)\cdot \text{cos}(\frac{2\pi \nu_{p}}{c}\cdot (z-z_{p})). \end{array}$$ (9)

Here *E*_*0*_ is the incident electric field strength amplitude, *H*_*0*_ is the incident magnetic field strength amplitude, *z*_*p*_ the incident pulse center position, *ν*_*p*_ and *ω*_*p*_ = 2*πν*_*p*_ are the carrier and angular pulse frequency, *a*_*z*_ is the pulse length in space, *τ*_*p*_ *= a*_*z*_*/c* is the pulse duration. The amplitudes for *E* and *H* are connected by well-known formulas (see below) at the electromagnetic wave propagation in a linear medium.

We suppose that the medium is in the ground state until an action of the THz pulse:

$$\rho_{11}=1,\;\rho_{mn}=0,\;m\neq 1,\;n\neq 1.$$ (10)

Obviously, this condition corresponds to the absolute zero value of temperature. Nevertheless, for the investigation of the cascade mechanism manifestation we consider “pure conditions”. Under room temperature, the effect will be still pronounced but its interpretation is difficult because a number of possible energy level transitions take place at the electromagnetic pulse action on the medium.

### 2.2 Dimensionless equations

For computer simulation, it is useful to transform the Eqs (1)–(10) to a dimensionless form. We use the following normalization, where the index “dim” means dimensionless variables:

$$z^{\text{dim}}=z\cdot \tau_{0}\cdot c,\;t^{\text{dim}}=t\cdot \tau_{0},\;\tau_{p}^{\text{dim}}=\tau_{0}\cdot \tau_{p},\;\omega_{p}^{\text{dim}}=\frac{1}{\tau_{0}}\cdot 2\pi \nu_{p},$$ (11)

$$d_{mn}^{\text{dim}}=\frac{\hbar }{E_{0}\tau_{0}}\cdot d_{mn},\;\omega_{mn}^{\text{dim}}=\frac{1}{\tau_{0}}\cdot \omega_{mn},\;\gamma_{mn}^{\text{dim}}=\frac{1}{\tau_{0}}\cdot \gamma_{mn},\;W_{mn}^{\text{dim}}=\frac{1}{\tau_{0}}\cdot W_{mn},$$ (12)

$$L_{l}^{\text{dim}}=L_{l}/c\tau_{0},\;L_{r}^{\text{dim}}=L_{r}/c\tau_{0},\;L_{t}^{\text{dim}}=L_{t}/c\tau_{0}.$$ (13)

Here *τ*_*0*_ is chosen to be equal to 10^−12^ s as a unit of time measurement. This value corresponds approximately to 0.3 mm. We also introduce the following dimensionless coefficient:

$$\kappa =\frac{M\hbar }{E_{0}^{2}\tau_{0}}=\frac{\left[cm^{-3}][cm^{2}g^{1}s^{-1}\right]}{[s^{1}]{\left[cm^{-0.5}g^{0.5}s^{-1}\right]}^{2}}\;(\text{CGS}).$$ (14)

Note that in the SI base units, this coefficient has the form [44]: $\kappa =\frac{M\hbar }{\varepsilon_{0}E_{0}^{2}\tau_{0}}=\frac{\left[m^{-3}][m^{2}kg^{1}s^{-1}\right]}{\left[m^{-3}kg^{-1}s^{4}A^{2}]{\left[m^{1}kg^{1}s^{-3}A^{-1}\right]}^{2}[s^{1}\right]}.$ For the dimensionless values of *E*, *H* and *P* we write the following relations:

$$E^{\text{dim}}=E\cdot E_{0},\;H^{\text{dim}}=H\cdot H_{0},\;P^{\text{dim}}=P\cdot E_{0}.$$ (15)

Using Eqs (11)–(15), Eqs (1)–(10) can be rewritten in the dimensionless form:

$$\frac{\partial H}{\partial t}=-\frac{\partial E}{\partial z},\;L_{l}<z\leq L_{r},$$ (16)

$$\frac{\partial D}{\partial t}=-\frac{\partial H}{\partial z},\;L_{l}<z\leq L_{r},$$ (17)

$$D=E,\;z\in [L_{l},z_{с1}]\cup [z_{с2},L_{R}],$$ (18)

$$D(z,t)=\varepsilon (z,t)\cdot E(z,t),\;z\in [z_{c1},z_{L}]\cup [z_{R},z_{c2}],$$ (19)

D=E+4πP, (20)

$$\frac{\partial \rho_{mn}}{\partial t}=-(\gamma_{mn}+i\omega_{mn})\rho_{mn}+iE\sum_{q=1}^{N}\left(d_{mq}\rho_{qn}-\rho_{mq}d_{qn}\right),$$ (21)

$$\frac{\partial \rho_{mm}}{\partial t}=-\sum_{q=1}^{N}\left(W_{mq}\rho_{mm}-W_{qm}\rho_{qq}\right)+iE\sum_{q=1}^{N}\left(d_{mq}\rho_{qm}-\rho_{mq}d_{qm}\right),$$ (22)

$$P=\kappa \sum_{n=1}^{N}\sum_{m=1}^{N}\left(d_{mn}\rho_{nm}\right)+\chi E,$$ (23)

$$z_{L}\leq z<z_{R},\;0<t\leq L_{t},\;n,m=\bar{1,N},$$ (24)

$$E(z,t=0)=\text{exp}(-{\left((z-z_{p})/\tau_{p}\right)}^{2})\cdot \text{cos}(\omega \cdot (z-z_{p})),$$ (25)

$$H(z,t=0)=\text{exp}(-{\left((z-z_{p})/\tau_{p}\right)}^{2})\cdot \text{cos}(\omega \cdot (z-z_{p})),$$ (26)

$$\rho_{11}(z,t=0)=1,\;\rho_{mn}(z,t=0)=0,\;m\neq 1,\;n\neq 1,\;z_{L}\leq z<z_{R}.$$ (27)

For brevity, an index "dim" is omitted below.

### 2.3 Invariants

#### 2.3.1 Maxwell’s equations invariants

To verify the computer simulation accuracy we follow the problem invariants, which can be easily obtained by integrating Eqs (16) and (17) along *z* and *t* coordinates.

After the integration, the following invariants can be written:

$$\int_{L_{l}}^{L_{r}}D(z,t)dz=I_{1}-\int_{0}^{t}\left(H(L_{r},t\prime )-H(L_{l},t\prime )\right)dt\prime ,$$ (28)

$$\int_{L_{l}}^{L_{r}}H(z,t)dz=I_{2}-\int_{0}^{t}\left(E(L_{l},t\prime )-E(L_{r},t\prime )\right)dt\prime .$$ (29)

Here, *I*_*1*_ and *I*_*2*_ are the constants determined by the incident pulse parameters.

If the electromagnetic field does not reach the computational domain boundaries, the invariants (28) and (29) are transformed:

$$\int_{L_{l}}^{L_{r}}D(z,t)dz=I_{1}=const,$$ (30)

$$\int_{L_{l}}^{L_{r}}H(z,t)dz=I_{2}=const.$$ (31)

#### 2.3.2 Bloch invariant

**Theorem 2.1.** *Provided relaxation rates W*_*mm*_ *and γ*_*mn*_ *are negligible*, *the density matrix evolution described by* Eqs (21) and (22) *obeys the following invariant*:

$$\sum_{m=2}^{N}\sum_{n=1}^{m-1}{\left(\rho_{mm}(z,t)-\rho_{nn}(z,t)\right)}^{2}+2N\sum_{m=2}^{N}\sum_{n=1}^{m-1}{\left|\rho_{mn}(z,t)\right|}^{2}=const(z).$$ (32)

**Proof.** Firstly, let us introduce auxiliary definitions:

$$\Omega_{mn}=-(\gamma_{mn}+i\omega_{mn}),\;m,n=\bar{1,N},$$ (33)

the purpose of which is to reduce the complexity of the forthcoming operations. Although we derive the invariant supposing the influence of relaxation determined by *γ*_*mn*_ negligible, it is still present in our consideration in order to demonstrate its role in invariant deterioration.

Secondly, let us remind that the matrices *d*, *ρ* and and Ω are Hermitian ones:

$$d_{mn}=d_{nm}^{*},\;\rho_{mn}=\rho_{nm}^{*},\;\Omega_{mn}=\Omega_{nm}^{*},\;m,n=\bar{1,N}.$$ (34)

Note here, that Ω_*mm*_ = 0, Ω_*mn*_ + Ω_*nm*_ = 2*γ*_*mn*_, because the matrices *ω* and *γ* are antisymmetric and symmetric real-valued matrices correspondingly, and the main diagonal elements of matrix *γ* is equal to zero:

$$\omega_{mn}=-\omega_{nm},\;\gamma_{mn}=\gamma_{nm},\;\gamma_{mm}=0,\;m,n=\bar{1,N}.$$ (35)

Using theses notations, Eqs (6) and (7) can be re-written in the following form:

$$\frac{\partial \rho_{mn}}{\partial t}=\Omega_{mn}\rho_{mn}+iE\sum_{q=1}^{N}\left(d_{mq}\rho_{qn}-d_{qn}\rho_{mq}\right),\;m,n=\bar{1,N}.$$ (36)

Let us further transform Eq (36) separating the terms in the sum, which contain diagonal and off-diagonal elements:

$$\frac{\partial \rho_{mn}}{\partial t}=\Omega_{mn}\rho_{mn}+\psi_{mn}+iE((d_{mm}-d_{nn})\rho_{mn}-d_{mn}\delta_{mn}),\;m,n=\bar{1,N}.$$ (37)

For brevity, we introduced above additional notations:

$$\psi_{mn}=iE\sum_{\begin{array}{l} q=1\\ q\neq m,n \end{array}}^{N}\left(d_{mq}\rho_{qn}-d_{qm}\rho_{nq}\right),\;\delta_{mn}=\rho_{mm}-\rho_{nn},\;m,n=\bar{1,N},$$ (38)

where *δ*_*mn*_ *= -δ*_*nm*_ is the difference of the energy levels populations. Now, we use well-known formula and Eq (35) to obtain the time derivative for the squared matrix elements, which are complex values:

$$\frac{\partial {\left|\rho_{mn}\right|}^{2}}{\partial t}=\frac{\partial (\rho_{mn}^{*}\rho_{mn})}{\partial t}=\rho_{mn}^{*}{\dot{\rho }}_{mn}+{\dot{\rho }}_{mn}^{*}\rho_{mn}=\rho_{nm}{\dot{\rho }}_{mn}+{\dot{\rho }}_{nm}\rho_{mn},\;m,n=\bar{1,N}.$$ (39)

Thus, multiplying Eq (37) by $\rho_{mn}^{*}$ and summing the result with its complex conjugate, and taking into account Eq (39), we obtain the following expression:

$$\begin{array}{ll} \frac{\partial {\left|\rho_{mn}\right|}^{2}}{\partial t} & =(\Omega_{mn}+\Omega_{nm})\rho_{mn}\rho_{nm}+\psi_{mn}\rho_{nm}+\psi_{nm}\rho_{mn}+iE((d_{mm}-d_{nn})+\\ & +(d_{nn}-d_{mm}))\rho_{mn}\rho_{nm}-iE(d_{mn}\delta_{mn}\rho_{nm}+d_{nm}\delta_{nm}\rho_{mn})=\\ & =-2\gamma_{mn}{\left|\rho_{mn}\right|}^{2}+\psi_{mn}\rho_{nm}+\psi_{nm}\rho_{mn}+\varphi_{mn},\;m,n=\bar{1,N}, \end{array}$$ (40)

Where

$$\varphi_{mn}=-iE(d_{mn}\delta_{mn}\rho_{nm}+d_{nm}\delta_{nm}\rho_{mn}),\;m,n=\bar{1,N}.$$ (41)

It is now seen from Eq (40) that the non-zero relaxation (*γ*_*mn*_*≠*0) causes exponential decay of each of terms in the second sum in the formula (32). Therefore, to derive the invariant, we assume below that *γ*_*mn*_ *=* 0. In general case, one can write a law of density matrix elements decaying.

Next, let us construct evolution equations for the squared population differences between two energy levels *δ*_*mn*_. After trivial transformations, it can be obtained from Eq (39) in the following form:

$$2\delta_{mn}\frac{\partial \delta_{mn}}{\partial t}=\frac{\partial \delta_{mn}{}^{2}}{\partial t}=2\delta_{mn}\sum_{\begin{array}{l} q=1\\ q\neq m,n \end{array}}^{N}\left(d_{mq}\rho_{qm}-d_{qm}\rho_{mq}-d_{nq}\rho_{qn}+d_{qn}\rho_{nq}\right)-4\varphi_{mn},\;m,n=\bar{1,N}.$$ (42)

Now, let us perform the summation of Eqs (40) at zero relaxation (*γ*_*mn*_ *=* 0) using Eq (38) and also the summation of Eq (42) for all the pairs of indices *m* and *n* such that 1≤*n*<*m*≤*N*:

$$\sum_{m=2}^{N}\sum_{n=1}^{m-1}\frac{\partial {\left|\rho_{mn}\right|}^{2}}{\partial t}=\sum_{m=2}^{N}\sum_{n=1}^{m-1}\left(iE\sum_{\begin{array}{l} q\neq m,n\\ q=1 \end{array}}^{N}(d_{mq}\rho_{qn}\rho_{nm}-d_{qn}\rho_{mq}\rho_{nm}+d_{nq}\rho_{qm}\rho_{mn}-d_{qm}\rho_{nq}\rho_{mn})+\varphi_{mn}\right),$$ (43)

$$\sum_{m=2}^{N}\sum_{n=1}^{m-1}\frac{\partial \delta_{mn}{}^{2}}{\partial t}=\sum_{m=2}^{N}\sum_{n=1}^{m-1}\left(2\delta_{mn}\sum_{\begin{array}{l} q\neq m,n\\ q=1 \end{array}}^{N}\left(d_{mq}\rho_{qm}-d_{qm}\rho_{mq}-d_{nq}\rho_{qn}+d_{qn}\rho_{nq}\right)-4\varphi_{mn}\right),\;m,n=\bar{1,N}.$$ (44)

We chose such summation limits using the analogy with the derivation of the Bloch invariant in the case of two energy levels medium and keep the time derivatives as separate sum for clearness of further transformations.

Let us notice that the sum $\overset{N}{\underset{m=2}{\sum }}\overset{m-1}{\underset{n=1}{\sum }}\overset{N}{\underset{\begin{array}{l} q\neq m,n\\ q=1 \end{array}}{\sum }}$ contains all combinations of three distinct integer numbers in the range $\bar{1,N}$. In combinatorics, the term “combination” means “a way to choose *k* distinct elements from a set of *l* elements without regard to the order of selection. And word “triple” below will be used to denote “a combination of three elements from the range $\bar{1,N}$”.

For each trio of distinct numbers, we can assign names *a*, *b*, *c* to them in such a way so that *a<b<c*. Let us now examine one such trios that is spanned according to the sum limits. There exists only three possible ways to assign numbers *a*, *b*, *c* to indices *m*, *n*, *q*. The possible assignments are listed in the Table 1.

**Table 1:** Possible assignments of specific values *a*, *b*, *c* to indices *m*, *n*, *q*.

*m*	*n*	*q*
*b*	*a*	*c*
*c*	*a*	*b*
*c*	*b*	*a*

Now let us examine the terms in Eq (43) related to the specific trio *a*, *b*, *c*:

$$\begin{array}{l} \sum_{m=2}^{N}\sum_{n=1}^{m-1}\frac{\partial {\left|\rho_{mn}\right|}^{2}}{\partial t}=\sum_{m=2}^{N}\sum_{n=1}^{m-1}\varphi_{mn}+\\ \begin{array}{l} +iE\sum_{m=2}^{N}\sum_{n=1}^{m-1}\sum_{\begin{array}{l} q\neq m,n\\ q=1 \end{array}}^{N}\left(d_{mq}\rho_{qn}\rho_{nm}-d_{qn}\rho_{mq}\rho_{nm}+d_{nq}\rho_{qm}\rho_{mn}-d_{qm}\rho_{nq}\rho_{mn}\right)+\\ m,n,q\neq b,a,c;\;\;\;m,n,q\neq c,a,b\;;\;\;\;m,n,q\neq c,b,a \end{array}\\ +iE(d_{bc}\rho_{ca}\rho_{ab}-d_{ca}\rho_{bc}\rho_{ab}+d_{ac}\rho_{cb}\rho_{ba}-d_{cb}\rho_{ac}\rho_{ba})+\\ +iE(d_{cb}\rho_{ba}\rho_{ac}-d_{ba}\rho_{cb}\rho_{ac}+d_{ab}\rho_{bc}\rho_{ca}-d_{bc}\rho_{ab}\rho_{ca})+\\ +iE(d_{ca}\rho_{ab}\rho_{bc}-d_{ab}\rho_{ca}\rho_{bc}+d_{ba}\rho_{ac}\rho_{cb}-d_{ac}\rho_{ba}\rho_{cb}){,}_{}{}_{}m,n=\bar{1,N}. \end{array}$$ (45)

The last three terms form the only possible set described by a particular combination of three numbers. These three terms cancel out each other for any arbitrary combination of numbers and the second sum in Eq (45) contains all such combinations. Thus, the second term in Eq (45) is equal to zero as well. Hence, the all-pairs sum of the squared off-diagonal elements is equal to:

$$\sum_{m=2}^{N}\sum_{n=1}^{m-1}\frac{\partial {\left|\rho_{mn}\right|}^{2}}{\partial t}=\sum_{m=2}^{N}\sum_{n=1}^{m-1}\varphi_{mn}.$$ (46)

Now, let us perform the same analysis for the population differences:

$$\begin{array}{l} \sum_{m=2}^{N}\sum_{n=1}^{m-1}\frac{\partial \delta_{mn}{}^{2}}{\partial t}=-4\sum_{m=2}^{N}\sum_{n=1}^{m-1}\varphi_{mn}+\\ \begin{array}{l} +2iE\sum_{m=2}^{N}\sum_{n=1}^{m-1}\sum_{\begin{array}{l} q\neq m,n\\ q=1 \end{array}}^{N}\left(d_{mq}\rho_{qm}\delta_{mn}-d_{qm}\rho_{mq}\delta_{mn}+d_{nq}\rho_{qn}\delta_{mn}-d_{qn}\rho_{nq}\delta_{mn}\right)+\\ m,n,q\neq b,a,c;\;\;\;m,n,q\neq c,a,b\;;\;\;\;m,n,q\neq c,b,a \end{array}\\ +2iE(d_{bc}\rho_{cb}\delta_{ba}-d_{cb}\rho_{bc}\delta_{ba}+d_{ac}\rho_{ca}\delta_{ba}-d_{ca}\rho_{ac}\delta_{ba})+\\ +2iE(d_{cb}\rho_{bc}\delta_{ca}-d_{bc}\rho_{cb}\delta_{ca}+d_{ab}\rho_{ba}\delta_{ca}-d_{ba}\rho_{ab}\delta_{ca})+\\ +2iE(d_{ca}\rho_{ac}\delta_{cb}-d_{ac}\rho_{ca}\delta_{cb}+d_{ba}\rho_{ab}\delta_{cb}-d_{ab}\rho_{ba}\delta_{cb}). \end{array}$$ (47)

Here it is important that definition of *δ*_*mn*_. Eq (38) provides that:

$$\delta_{ab}+\delta_{bc}=\delta_{ac}.$$ (48)

Taking into account Eq (48) along with the definition (41), the Eq (47) can be transformed into:

$$\begin{array}{l} \sum_{m=2}^{N}\sum_{n=1}^{m-1}\frac{\partial \delta_{mn}{}^{2}}{\partial t}=-4\sum_{m=2}^{N}\sum_{n=1}^{m-1}\varphi_{mn}+\\ \begin{array}{l} +2iE\sum_{m=2}^{N}\sum_{n=1}^{m-1}\sum_{\begin{array}{l} q\neq m,n\\ q=1 \end{array}}^{N}\left(d_{mq}\rho_{qm}\delta_{mn}-d_{qm}\rho_{mq}\delta_{mn}+d_{nq}\rho_{qn}\delta_{mn}-d_{qn}\rho_{nq}\delta_{mn}\right)-2(\varphi_{ba}+\varphi_{ca}+\varphi_{cb}).\\ m,n,q\neq b,a,c;\;\;\;m,n,q\neq c,a,b\;;\;\;\;m,n,q\neq c,b,a \end{array} \end{array}$$ (49)

We see that our analysis demonstrates the appearance of an additional term −2(*φ*_*ba*_ + *φ*_*ca*_ + *φ*_*cb*_) in Eq (49), which contains *φ*_*mn*_ indexed by all three possible two number combinations in the triple *a*, *b*, *c*. There are $\left(\begin{array}{c} N\\ 3 \end{array}\right)$ of various triples that the second term contains, in these combinations of three elements each possible combination of two elements is repeated *N-*2 times in total. So, *φ*_*mn*_ associated with each particular pair of indices appears in the sum *N-*2 times as well and Eq (49) can be transformed into:

$$\sum_{m=2}^{N}\sum_{n=1}^{m-1}\frac{\partial \delta_{mn}{}^{2}}{\partial t}=-4\sum_{m=2}^{N}\sum_{n=1}^{m-1}\varphi_{mn}-2(N-2)\sum_{m=2}^{N}\sum_{n=1}^{m-1}\varphi_{mn},$$ (50)

which is simplified further:

$$\sum_{m=2}^{N}\sum_{n=1}^{m-1}\frac{\partial \delta_{mn}{}^{2}}{\partial t}=-2N\sum_{m=2}^{N}\sum_{n=1}^{m-1}\varphi_{mn}.$$ (51)

Now it is evident, that we can obtain the required invariant in time (32) by multiplying Eq (46) by 2*N* and summing it with Eq (51).

## 3. Finite-difference scheme

To solve Eqs (16)–(27), we use a combination of the well-known Yee’s scheme [36], [45] for Maxwell’s equations and a Crank-Nicolson scheme for the equations with respect to the density matrix with an iteration process. Therefore, to realize the positiveness property of the density matrix diagonal elements we use a sufficiently small mesh steps in computer simulation.

### 3.1 Approximation of the equations

The model (16)–(27) is approximated on two offset grids Ω = *ω*_*z*_ × *ω*_*t*_ and $\tilde{\Omega }=\tilde{\omega_{z}}\times \tilde{\omega_{t}}$ in the domain *G* = [0, *L*_*t*_] × [*L*_*l*_, *L*_*r*_].

$$\omega_{t}=\left\{t_{l}=l\cdot \tau ,\;l=\bar{0,N_{t}},\;\tau =\frac{L_{t}}{N_{t}}\right\},$$ (52)

$${\tilde{\omega }}_{t}=\left\{t_{l+0.5}=(l+0.5)\cdot \tau ,\;l=\bar{0,N_{t}-1},\;\tau =\frac{L_{t}}{N_{t}}\right\},$$ (53)

$$\omega_{z}=\left\{z_{j}=j\cdot h-L_{l},\;j=\bar{0,N_{z}},\;h=\frac{L_{z}}{N_{z}}\right\},$$ (54)

$${\tilde{\omega }}_{z}=\left\{z_{j+0.5}=(j+0.5)\cdot h-L_{l},\;j=\bar{0,N_{z}-1},\;h=\frac{L_{z}}{N_{z}}\right\}.$$ (55)

Here *N*_*z*_ and *N*_*t*_ are numbers of nodes along spatial coordinate and time, correspondingly.

Let us also introduce the following notations for nodes:

$$j_{p}=[(z_{p}-L_{l})/h],\;j_{c1}=[(z_{c1}-L_{l})/h],\;j_{c2}=[(z_{c2}-L_{l})/h].$$ (56)

$$j_{L}=[(z_{L}-L_{l})/h],\;j_{R}=[(z_{R}-L_{l})/h],\;n_{l}=[h_{l}/h_{z}],$$ (57)

which correspond to the incident pulse centre coordinate; the coordinates of the disordered structure beginning and ending and coordinates of the medium beginning and ending and the number of nodes inside single cover layer, correspondingly (see Fig 1). The square brackets in Eqs (56) and (57) mean the integer part of the fraction. (We always use parameters such that these indices are integer without rounding.)

The mesh function *ε*(*z*_*j*_) for the dielectric permittivity is defined on the grid *ω*_*z*_:

$$\begin{array}{l} \varepsilon (z_{j})=\{\begin{array}{c} (1+\varepsilon_{J_{c1}})/2,\;\;j=j_{c1},\\ \varepsilon_{J},\;\;j=\bar{\left(j_{c1}+(J-1)n_{l}+1),\;\;(j_{c1}+J\cdot n_{l}-1\right)},\;J=\bar{1,N_{l}},\\ {\tilde{\varepsilon }}_{J},\;\;j=\bar{\left(j_{R}+(J-1)n_{l}+1),\;(j_{R}+J\cdot n_{l}-1\right)},\;J=\bar{1,N_{l}},\\ (\varepsilon_{J}+\varepsilon_{J+1})/2,\;\;j=j_{c1}+J\cdot n_{l},\;J=\bar{1,N_{l}-1,}\\ ({\tilde{\varepsilon }}_{J}+{\tilde{\varepsilon }}_{J+1}))/2,\;\;j=j_{R}+J\cdot n_{l},\;J=\bar{1,N_{l}-1,}\\ ({\tilde{\varepsilon }}_{J_{c2}}+1)/2,\;\;j=j_{c2}. \end{array}\\ \; \end{array}$$ (58)

We note that the dielectric permittivity is defined in the same nodes as the electric field induction and electric field strength. Mesh function for the magnetic field strength is defined on the grid $\tilde{\Omega }:H(z_{j+0.5},t_{l+0.5})$. The mesh functions for the electric field strength and its induction as well as for the mesh functions for density matrix elements and medium polarization are defined on the grid Ω: *D*(*z*_*j*_, *t*_*l*_), *E*(*z*_*j*_, *t*_*l*_), *ρ*_*mn*_(*z*_*j*_, *t*_*i*_), *P*(*z*_*j*_, *t*_*l*_), correspondingly. For brevity, we save the same notations for the mesh functions as for the differential functions introduced above.

Eqs (16) and (17) are approximated using the Yee’s method. Therefore, in our notations the corresponding finite-difference scheme is written as

$$\frac{H(z_{j+0.5},t_{l+0.5})-H(z_{j+0.5},t_{l-0.5})}{\tau }=-\frac{E(z_{j+1},t_{l})-E(z_{j},t_{l})}{h},$$ (59)

$$\frac{D(z_{j},t_{l+1})-D(z_{j},t_{l})}{\tau }=-\frac{H(z_{j+0.5},t_{l+0.5})-H(z_{j-0.5},t_{l+0.5})}{h},\;l=\bar{0,N_{t}-1},\;j=\bar{1,N_{z}-1}.$$ (60)

In vacuum, the electric field strength is equal to its induction:

$$D(z_{j},t_{l})=E(z_{j},t_{l}),\;l=\bar{0,N_{t}},\;j=\bar{1,j_{c1}-1}\cup \bar{j_{c2}+1,N_{z}}-1,$$ (61)

and in the cover layers their relation is defined as:

$$E(z_{j},t_{l})=D(z_{j},t_{l})/\varepsilon (z_{j}),\;l=\bar{0,N_{t}},\;j=\bar{j_{c1},j_{L}-1}\cup \bar{j_{R}+1,j_{c2}}.$$ (62)

A relation between the mesh functions for the electric field strength and its induction for the medium is written below after writing the finite-difference scheme for the density matrix.

The novel feature of this well-known finite-difference scheme consists of the multilevel Maxwell-Bloch equations approximation for a medium response description. So, the approximation of Eqs (20)–(23), based on the Crank-Nicolson scheme, is made in the following form:

$$\frac{\rho_{mn}(z_{j},t_{l+1})-\rho_{mn}(z_{j},t_{l})}{\tau }+(\gamma_{mn}+i\omega_{mn}){\tilde{\rho }}_{mn}=i\tilde{E}\cdot \sum_{q=1}^{N}\left(d_{mq}{\tilde{\rho }}_{qn}-{\tilde{\rho }}_{mq}d_{qn}\right),$$ (63)

$$\frac{\rho_{mm}(z_{j},t_{l+1})-\rho_{mm}(z_{j},t_{l})}{\tau }=-\sum_{q=1}^{N}\left(W_{mq}{\tilde{\rho }}_{mm}-{\tilde{\rho }}_{qq}W_{qm}\right)+i\tilde{E}\cdot \sum_{q=1}^{N}\left(d_{mq}{\tilde{\rho }}_{qm}-{\tilde{\rho }}_{mq}d_{qm}\right),$$ (64)

$$\tilde{E}=0.5\left(E(z_{j},t_{l+1})+E(z_{j},t_{l})\right),\;{\tilde{\rho }}_{mn}=0.5\left(\rho_{mn}(z_{j},t_{l+1})+\rho_{mn}(z_{j},t_{l})\right),$$ (65)

$$P(z_{j},t_{l+1})=\chi E(z_{j},t_{l+1})+\kappa \sum_{q=1}^{N}\sum_{r=1}^{N}d_{qr}\rho_{rq}(z_{j},t_{l+1}),\;j=\bar{j_{L},j_{R}},$$ (66)

$$E(z_{j},t_{l+1})=D(z_{j},t_{l+1})-4\pi P(z_{j},t_{l+1}),\;j=\bar{j_{L}+1,j_{R}-1},\;l=\bar{0,N_{t}-1},$$ (67)

$$D(z_{j_{L}},t_{l})=(\varepsilon_{N_{l}}E(z_{j_{L}-1},t_{l})+E(z_{j_{L}+1},t_{l})+\kappa P(z_{j_{L}+1},t_{l}))/2,$$ (68)

$$D(z_{j_{R}},t_{l})=({\tilde{\varepsilon }}_{N_{l}}E(z_{j_{R}+1},t_{l})+E(z_{j_{R}-1},t_{l})+\kappa P(z_{j_{R}-1},t_{l}))/2.$$ (69)

### 3.2 Boundary conditions approximation

At the edges of the computational domain, Eqs (1) and (2) are replaced with equations supplying a one-directional propagation, which makes boundaries transparent and reflectionless.

In the boundary node *j = N*_*z*_ we use the Cabaret approximation [42] for the translation operator:

$$\begin{array}{c} \frac{1}{2}\left(\frac{H(z_{N_{z}-0.5},t_{l+0.5})-H(z_{N_{z}-0.5},t_{l-0.5})}{\tau }+\frac{H(z_{N_{z}-1.5},t_{l-0.5})-H(z_{N_{z}-1.5},t_{l-1.5})}{\tau }\right)+\\ +\left(\frac{H(z_{N_{z}-0.5},t_{l-0.5})-H(z_{N_{z}-1.5},t_{l-0.5})}{h}\right)=0,\;l=\bar{2,\;N_{t}-1}, \end{array}$$ (70)

$$\frac{1}{2}\left(\frac{D(z_{{\text{N}}_{\text{z}}},t_{l+1})-D(z_{N_{z}},t_{l})}{\tau }+\frac{D(z_{N_{z}-1},t_{l})-D(z_{N_{z}-1},t_{l-1})}{\tau }\right)+\frac{D(z_{N_{z}},t_{l})-D(z_{N_{z}-1},t_{l})}{h}=0.$$ (71)

The corresponding difference equation for the first node on the spatial mesh is written in the following form:

$$\frac{1}{2}\left(\frac{H(z_{0.5},t_{l+0.5})-H(z_{0.5},t_{l-0.5})}{\tau }+\frac{H(z_{1.5},t_{l-0.5})-H(z_{1.5},t_{l-1.5})}{\tau }\right)-\frac{H(z_{1.5},t_{l-0.5})-H(z_{0.5},t_{l-0.5})}{h}=0,$$ (72)

$$\frac{1}{2}\left(\frac{D(z_{\text{0}},t_{l+1})-D(z_{0},t_{l})}{\tau }+\frac{D(z_{1},t_{l})-D(z_{1},t_{l-1})}{\tau }\right)-\frac{D(z_{1},t_{l})-D(z_{0},t_{l})}{h}=0,\;l=\bar{2,\;N_{t}-1}.$$ (73)

### 3.3 Initial condition approximation

We see that these Eqs (70)–(73) are not applicable on the first time step *l* = 1. Since we consider the finite incident electromagnetic pulse, therefore we can define the initial mesh functions for the electromagnetic field distribution in the following way:

$$E(z_{j},t_{0})=E_{0}\cdot \text{exp}(-{\left(z_{j}-hj_{p}\right)}^{2}/\tau_{p}^{2})\cdot \text{cos}(\omega_{p}\cdot (z_{j}-hj_{p})),\;j=\bar{2,N_{z}-2},$$ (74)

$$H(z_{j+0.5},t_{0.5})=E_{0}\cdot \text{exp}(-{\left(z_{j+05}-hj_{p}\right)}^{2}/\tau_{p}^{2})\cdot \text{cos}(\omega_{p}\cdot (z_{j+0.5}-hj_{p})),\;j=\bar{2,N_{z}-3},$$ (75)

$$\begin{array}{c} E(z_{j},t_{0})=0,\;j=0,1,N_{z}-1,N_{z},\\ H(z_{j+0.5},t_{0.5})=0,\;j=0,1,N_{z}-2,N_{z}-1. \end{array}$$ (76)

Here *j*_*p*_ is the mesh node index corresponding to the position of the initial pulse center:

$$j_{p}=(z_{p}-L_{l})/h.$$ (77)

Note that the initial values of *H* are defined at *t*_0.5_ node, which corresponds to the propagation of a wave packet in positive direction of *z*-coordinate. Therefore, taking into account the wave equation we can define the zero-value mesh functions in the boundary nodes on the second time layer (*l* = 1):

$$E(z_{1},t_{1})=E(z_{N_{z}},t_{1})=H(z_{N_{z}-0.5},t_{1.5})=H(z_{0.5},t_{1.5})=0.$$ (78)

The initial state of the medium is approximated in the following way:

$$\rho_{11}(z_{j},t_{0})=1,$$ (79)

$$\rho_{mn}(z_{j},t_{0})=0,\;\forall m\neq 1,\forall n\neq 1,\;j=\bar{j_{L},j_{R}}.$$ (80)

### 3.4 Proof of finite-difference scheme conservativeness

**Theorem 3.1.** *The finite-difference scheme* (58)–(80) *is conservative one and preserves difference analogues of the invariants* (28)–(31).

**Proof.** The invariants can be obtained by performing the summation of Eqs (59) and (60) with taking into account the boundary conditions (70)–(73). After that, we get the conservation law in the form:

$$\begin{array}{l} \sum_{j=0}^{N_{z}-1}(H(z_{j+0.5},t_{N_{t}-0.5})-H(z_{j+0.5},t_{1.5}))=-\frac{\tau }{h}\sum_{l=2}^{N_{t}-1}(E(z_{N_{z}-1},t_{l})-E(z_{1},t_{l}))+\\ +2\frac{\tau }{h}\sum_{l=2}^{N_{t}-1}\left(H(z_{1.5},t_{l-0.5})-H(z_{0.5},t_{l-0.5})\right)-(H(z_{1.5},t_{N_{t}-1.5})-H(z_{1.5},t_{0.5}))-\\ -2\frac{\tau }{h}\sum_{l=2}^{N_{t}-1}\left(H(z_{N_{z}-0.5},t_{l-0.5})-H(z_{N_{z}-1.5},t_{l-0.5})\right)-(H(z_{N_{z}-0.5},t_{N_{t}-1.5})-H(z_{N_{z}-1.5},t_{0.5})). \end{array}$$ (81)

Applying a similar procedure to the Eq (60), we obtain:

$$\begin{array}{l} \sum_{j=0}^{N_{z}-1}(D(z_{j},t_{N_{t}})-D(z_{j},t_{0}))=-\frac{\tau }{h}\sum_{l=2}^{N_{t}-1}(H(z_{N_{z}-0.5},t_{l+0.5})-H(z_{0.5},t_{l+0.5}))+\\ +2\frac{\tau }{h}\sum_{l=2}^{N_{t}-1}\left(D(z_{1},t_{l-1})-D(z_{0},t_{l-1})\right)-(D(z_{1},t_{N_{t}-2})-D(z_{1},t_{0}))-\\ -2\frac{\tau }{h}\sum_{l=2}^{N_{t}-1}\left(D(z_{N_{z}-1},t_{l-1})-D(z_{N_{z}-2},t_{l-1})\right)-(D(z_{N_{z}-1},t_{N_{t}-2})-D(z_{N_{z}-2},t_{0})). \end{array}$$ (82)

If there are the zero-value boundary conditions for the electromagnetic field then Eqs (81) and (82) transform into the equalities:

$$\sum_{j=0}^{N_{z}}D(z_{j},t_{l+1})=\sum_{j=0}^{N_{z}}D(z_{j},t_{0})=const,\;l=\bar{0,N_{t}-1},$$ (83)

$$\sum_{j=0}^{N_{z}-1}H(z_{j+0.5},t_{l+0.5})=\sum_{j=0}^{N_{z}-1}H(z_{j+0.5},t_{0.5})=const,\;l=\bar{0,N_{t}-1}.$$ (84)

**Theorem 3.2.** *A difference analogue of the density matrix evolution invariant* (32) *holds in each node of the grid* Ω:

$$\sum_{m=2}^{N}\sum_{n=1}^{m-1}{\left(\rho_{mm}(z_{j},t_{l})-\rho_{nn}(z_{j},t_{l})\right)}^{2}+2N\sum_{m=2}^{N}\sum_{n=1}^{m-1}{\left|\rho_{mn}(z_{j},t_{l})\right|}^{2}=const(z_{j}).$$ (85)

$$j=\bar{j_{L},j_{R}},\;l=\bar{0,N_{t}-1}.$$

**Proof.** The proof repeats most steps from the same invariant derivation in the differential form (see Theorem 2.1). It starts from the Eqs (63) and (64). Similar to Eqs (33), (38) and (41) we introduce the following notations:

$$\begin{array}{c} {\tilde{\psi }}_{mn}=i\tilde{E}\sum_{\begin{array}{l} q=1\\ q\neq m,n \end{array}}^{N}\left(d_{mq}{\tilde{\rho }}_{qn}-d_{qn}{\tilde{\rho }}_{mq}\right),\;{\tilde{\delta }}_{mn}={\tilde{\rho }}_{mm}-{\tilde{\rho }}_{nn},\\ {\tilde{\varphi }}_{mn}=-i\tilde{E}(d_{mn}{\tilde{\delta }}_{mn}{\tilde{\rho }}_{nm}+d_{nm}{\tilde{\delta }}_{nm}{\tilde{\rho }}_{mn}). \end{array}$$ (86)

Definitions of $\tilde{E}$ and $\tilde{\rho }$ are the same as in Eq (65). With these notations, the Eqs (63) and (64) are written as:

$$\frac{\rho_{mn}(z_{j},t_{l+1})-\rho_{mn}(z_{j},t_{l})}{\tau }=\Omega_{mn}{\tilde{\rho }}_{mn}+i\tilde{E}\sum_{q=1}^{N}\left(d_{mq}{\tilde{\rho }}_{qn}-{\tilde{\rho }}_{mq}d_{qn}\right).$$ (87)

Next, we write the squared derivatives of the non-diagonal elements in the following way:

$$\begin{array}{c} \frac{{\left|\rho_{mn}(z_{j},t_{l+1})\right|}^{2}-{\left|\rho_{mn}(z_{j},t_{l})\right|}^{2}}{\tau }=\\ ={\tilde{\rho }}_{mn}^{*}\frac{\rho_{mn}(z_{j},t_{l+1})-\rho_{mn}(z_{j},t_{l})}{\tau }+\frac{\rho_{mn}^{*}(z_{j},t_{l+1})-\rho_{mn}^{*}(z_{j},t_{l})}{\tau }{\tilde{\rho }}_{mn}. \end{array}$$ (88)

Using this definition, we repeat steps (34) and (40) of the analytical derivation and obtain an analogue of Eq (43):

$$\frac{{\left|\rho_{mn}(z_{j},t_{l+1})\right|}^{2}-{\left|\rho_{mn}(z_{j},t_{l})\right|}^{2}}{\tau }=2\gamma_{mn}{\left|{\tilde{\rho }}_{mn}\right|}^{2}+{\tilde{\psi }}_{mn}{\tilde{\rho }}_{nm}+{\tilde{\psi }}_{nm}{\tilde{\rho }}_{mn}+\varphi_{mn}.$$ (89)

Similarly, we obtain the analog of the Eq (42) for the ${\tilde{\delta }}_{mn}$:

$$\frac{{\left|\delta_{mn}(z_{j},t_{l+1})\right|}^{2}-{\left|\delta_{mn}(z_{j},t_{l})\right|}^{2}}{\tau }=2{\tilde{\delta }}_{mn}\sum_{q\neq m,n}\left(d_{mq}{\tilde{\rho }}_{qm}-d_{qm}{\tilde{\rho }}_{mq}-d_{nq}{\tilde{\rho }}_{qn}+d_{qn}{\tilde{\rho }}_{nq}\right)-4{\tilde{\varphi }}_{mn}.$$ (90)

Next, repeating the steps (42)–(51) of the analytical derivation one can easily obtain an analogue of the invariant (32):

$$\sum_{m=2}^{N}\sum_{n=1}^{m-1}{\left(\rho_{mm}(z_{j},t_{l})-\rho_{nn}(z_{j},t_{l})\right)}^{2}+2N\sum_{m=2}^{N}\sum_{n=1}^{m-1}{\left|\rho_{mn}(z_{j},t_{l})\right|}^{2}=const(z_{j}).$$ (91)

### 3.5 Approximation order investigation

**Theorem 3.3.** *The finite-difference scheme* (59)–(73) *approximates the set of differential* Eqs (16)–(23) *with the second order in the inner nodes of the mesh*. *Namely*, *the* Eq (59) *approximates the* Eq (16) *with respect to the point* (*z*_*j+0*.*5*_, *t*_*l*_). *The* Eq (60) *approximates the* Eq (17) *with respect to the point* (*z*_*j*_, *t*_*l+0*.*5*_). *The density matrix* Eqs (21)–(23) *are approximated by* (63)–(67) *with the second order with respect to the point* (*z*_*j*_, *t*_*l+0*.*5*_).

The theorem 3.3 is proved in a standard way with the help of the Taylor series representation.

The boundary conditions are approximated by the Cabaret finite-difference scheme (70)–(73) with the second order in the points $(z_{1},t_{l-0.5}),\;(z_{0.5},t_{l}),\;(z_{N_{z}-0.5},t_{l}),\;(z_{N_{z}-1},t_{l-0.5}),\;l=\bar{2,N_{t}-1}.$.

We stress that the difference analogues of the invariants (82), (83) and (86) preserve their values during the computation time with the accuracy not worse than 10^−6^ for the mesh steps being equal to 0.01 (the full set of parameters are shown in the next section). Invariant (86) takes place only in the case of zero relaxation rate of off-diagonal elements. In our simulation, relaxation is non-negligible and therefore, the invariant value decreases over time as expected. In current paper, we have to introduce this invariant to explain peculiarities in population dynamics (section 2.3.2).

### 3.6 Iterations convergence proving

The difference Eqs (59)–(69) are nonlinear ones in respect to the electric field strength *E* and the density matrix elements *ρ*_*mn*_. To solve the equations, the following iteration method is used:

$$\frac{\rho_{mn}^{s+1}(z_{j},t_{l+1})-\rho_{mn}(z_{j},t_{l})}{\tau }=-(\gamma_{mn}+i\omega_{mn}){\tilde{\rho }}_{mn}^{s}+i{\tilde{E}}^{s}\cdot \sum_{q=1}^{N}\left(d_{mq}{\tilde{\rho }}_{qn}^{s}-{\tilde{\rho }}_{mq}^{s}d_{qn}\right),$$ (92)

$$\frac{\rho_{mm}^{s+1}(z_{j},t_{l+1})-\rho_{mm}(z_{j},t_{l})}{\tau }=-\sum_{q=1}^{N}(W_{mq}{\tilde{\rho }}_{mm}^{s}-{\tilde{\rho }}_{qq}^{s}W_{qm})+i{\tilde{E}}^{s}\cdot \sum_{q=1}^{N}\left(d_{mq}{\tilde{\rho }}_{qm}^{s}-{\tilde{\rho }}_{mq}^{s}d_{qm}\right),$$ (93)

$$E^{s+1}(z_{j},t_{l+1})=\frac{D(z_{j},t_{l+1})-4\pi \kappa \sum_{m=1}^{N}\sum_{n=1}^{N}d_{mn}\rho_{nm}^{s}(z_{j},t_{l+1})}{4\pi \chi +1},$$ (94)

$${\tilde{E}}^{s}=0.5\left(E^{s}(z_{j},t_{l+1})+E(z_{j},t_{l})\right),\;{\tilde{\rho }}_{mn}^{s}=0.5\left(\rho_{mn}^{s}(z_{j},t_{l+1})+\rho_{mn}(z_{j},t_{l})\right),\;s=1,2,\ldots ,$$ (95)

$$j=\bar{j_{L},j_{R}},\;l=\bar{0,N_{t}-1}.$$

The functions on zero-iteration are chosen equal to their values on the previous time layer:

$$E^{0}(z_{j},t_{l+1})=E(z_{j},t_{l}),\;\rho_{qm}^{0}(z_{j},t_{l+1})=\rho_{qm}(z_{j},t_{l}),\;j=\bar{j_{L},j_{R}},\;l=\bar{0,N_{t}-1}.$$ (96)

The iteration process is stopped if both of the following conditions are satisfied:

$$\left|E^{s+1}(z_{j},t_{l+1})-E^{s}(z_{j},t_{l+1})\right|<e_{1}\left|E^{s}(z_{j},t_{l+1})\right|+e_{2},$$ (97)

$$\left|\rho_{mn}^{s+1}(z_{j},t_{l+1})-\rho_{mn}^{s}(z_{j},t_{l+1})\right|<e_{1}\left|\rho_{mn}^{s}(z_{j},t_{l+1})\right|+e_{2},$$ (98)

$$j=\bar{j_{L},j_{R}},\;l=\bar{0,N_{t}-1}.$$ (99)

where *e*_1_ and *e*_2_ are positive constants, characterizing the maximal absolute and relative accuracy of the iteration process. In our computer simulations, their values were chosen as 10^−8^ and 10^−10^ correspondingly.

The procedure for the solution of the system (59)–(62) and (92)–(95) is the following. First, from Eq (59) we obtain the value of *H*(*z*_*j*+0.5_, *t*_*l*+0.5_) on the next time layer, then we have *D*(*t*_*l*+1_, *z*_*j*+1_) from Eq (60) and *E*^*s+*1^(*z*_*j*_, *t*_*l*+1_) from Eq (94). We substitute the last value in the Eqs (92) and (93) at the next iteration step, etc.

**Theorem 3.4**. *Provided that h<const and zero-iterations are chosen as* (96), *the process of iterations* (92)–(95) *converges to the unique solution of the finite-difference scheme* (59)–(69), *which exists in a vicinity of the mesh functions defined by the zero iteration* (96). *Convergence rate is as a geometric progression with the denominator q≈h*.

**Proof.** To prove this theorem, we have to demonstrate that (92)–(95) is a contraction, i.e. that all iterations are uniformly limited in a certain difference norm ‖⋅‖_*h*_:

$$||E^{s}|{|}_{h}\leq C_{E},\;||\rho^{s}|{|}_{h}\leq C_{\rho },$$ (100)

where *C*_*E*_ and *C*_*ρ*_ are constants, and that the following inequalities:

$$\begin{array}{c} ||E^{s+1}-E^{s}|{|}_{h}\leq q||E^{s}-E^{s-1}|{|}_{h},\\ ||\rho^{s+1}-\rho^{s}|{|}_{h}\leq q||\rho^{s}-\rho^{s-1}|{|}_{h},\;q<1 \end{array}$$ (101)

are valid. We use the difference norm *C*:

$$||f|{|}_{C}=\underset{z_{j}}{\text{max}}(|f(z_{j})|).$$ (102)

In Eq (102) the letter "*f*" denotes mesh function defined on the mesh. We estimate the function modules on different iteration steps and show that they are uniformly bounded in the norm (102). Then considering the iteration differences ‖*E*^*s*+1^ − *E*^*s*^‖_*C*_ and ‖*ρ*^*s*+1^ − *ρ*^*s*^‖_*C*_, and using estimations similar to those applied for the iteration modules we get the inequalities (102) in the norm (102).

### 3.7 Numerical stability of the finite-difference scheme

A stability condition for the finite-difference scheme (59)–(69) is the Courant-Friedrichs-Levy (CFL) condition [46], because the developed scheme involves the Yee’s one. When the Maxwell’s equations are solved, the CFL condition is well-known for the linear wave equation and has the form *u ≤ h/τ*, where *u* is the wave propagation velocity [46]. In Eqs (59) and (60) the wave velocity *u* for a linear medium with *ε* = 1 (vacuum) is equal to unity (*u =* 1), the mesh steps are chosen to be equal: *h = τ*, and the CFL condition reduces to the inequality *u ≤* 1. Thus, for the wave propagation in a vacuum the CFL condition is valid: *u =* 1. In the active medium as well as in the disordered structure, the wave velocity is less than unity because the dielectric permittivity is greater than unity and $u=1/\sqrt{\varepsilon }$ in the dimensionless units. Therefore, the CFL condition is also fulfilled.

It should be stressed that the preservation of the invariants (Theorem 3.1) is an additional confirmation of the solution correctness.

## 4. Computer simulation results

The aim of investigation provided below consists in an analysis of the pulses reflected from and transmitted through a three energy-level medium covered by disordered structure. We analyze the pulse structure (it consists from several sub-pulses) and its spectrum, and the spectra of sub-pulses. We pay attention to the substance emission at the transition 3–1 (high frequency), which appears due to the cascade mechanism of high energy level excitation and we demonstrate the evidence of its manifestation.

### 4.1 Computer simulation parameters

The computer simulation results for substance with covering are averaged over 32 random realizations for the cover dielectric permittivity random distribution. Usually, in physical experiments [29] we made also 16 or 32 realizations at the measurements of THz signals with duration about 100 ps. If we use more random realizations, then the measurement time increases up to 1 min, which is not acceptable in practice.

The electric field strength unit *E*_0_ was chosen to be 1.05·10^6^ V/m. Using the well-known formula, we can estimate the peak intensity of the pulse in SI units as

$$I=с\varepsilon_{0}E_{0}^{2}.$$ (103)

This formula yields the maximal intensity of the pulse equal to 3·10^5^ W/cm^2^. Another well-known formula [46] gives the value of the magnetic field induction and the magnetic field strength:

$$\left|B_{0}\right|=\frac{n}{c}\left|E_{0}\right|,\;\sqrt{\frac{\mu_{o}}{\varepsilon_{0}}}\left|H_{0}\right|=n\left|E_{0}\right|,$$ (104)

where $n=\sqrt{\varepsilon \mu }$ is the refractive index. Taking into account that in a vacuum *ε = μ =* 1, we obtain: *B*_0_ *=* 3.5·10^−3^ T, *H*_0_ *=* 2785 A/m (or 27.85 A/cm). Evidently, for practice the intensity less than 10^6^ W/cm^2^ is more reasonable but in computer simulation, the effect of second harmonic generation is better pronounced for greater than this value of the pulse intensity.

The dipole moment unit *d*_0_ is equal [47] to 1.47·10^−18^ cm^2.5^g^0.5^s^-1^ or 4.58·10^−30^ C·m. The density of the molecules in the medium is equal to *M* = 5.67·10^21^ cm^-3^ = 5.67·10^27^ m^-3^. In this case the coefficient *κ* = 12.5 in the Eq (15).

We chose the mesh steps as *h = τ =* 0.01. Iteration process parameters are chosen as *e*_1_ = 10^−8^ and *e*_1_ = 10^−10^, their decreasing does not result in notable changes of the computer simulation results at chosen mesh steps. Other dimensionless coordinates are given in the Table 2. The parameters of the medium and the pulse are provided in the Table 3.

**Table 2:** The dimensionless coordinates used in the computations.

*L*_*l*_ = −14	*L*_*r*_ = 34	*z*_*c*1_ = 0	*z*_*L*_ = 5	*z*_*R*_ = 11
*z*_*c*2_ = 16	*z*_*refl*_ = −13.9	*z*_*tran*_ = 17	*z*_*p*_ = −7	*L*_*t*_ = 100

**Table 3:** The dimensionless parameters of the medium and the pulse.

$d_{mn}=\left(\begin{array}{ccc} 0 & 1 & 9\\ 1 & 0 & 9\\ 1 & 9 & 0 \end{array}\right)\cdot 3.4\cdot 10^{-3}\;$	$\gamma_{mn}=\left(\begin{array}{ccc} 0 & 2 & 2\\ 2 & 0 & 1\\ 2 & 18 & 0 \end{array}8\right)$	$\omega_{mn}=\left(\begin{array}{ccc} 0 & -5 & -11\\ 5 & 0 & -6\\ 11 & 6 & 0 \end{array}\right)$
*W*_*mn*_ = 0	*L*_*s*_ = 6	*κ* = 12.5
*ω*_*p*_ = 5	*a*_*z*_ = 1.5	*χ* = 3
*h*_*l*_ = 0.25	*N*_*l*_ = 20	*ε*_*l*_ = [1, 3]

### 4.2 Transmitted and reflected pulse structure

**Fig 2:**
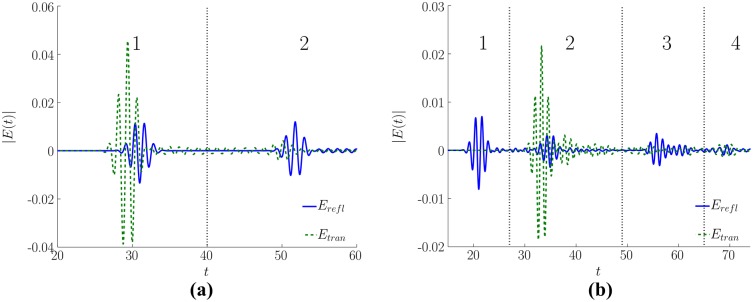
Pulses *E*_*refl*_ and *E*_*tran*_ reflected from (solid line) and transmitted through (dashed line) the medium without cover (a), and with cover (b) under averaging over 32 random realizations of disordered structure (dimensionless units).

The computer simulation is made for two cases: with the cover presence and without it to clarify its influence on the reflected and transmitted pulse spectra. The typical signals are depicted in Fig 2. One can see that in both cases the spatial pulse length is comparable with the length of a medium. The pulse splitting into a few sub-pulses is clearly seen for both covered and uncovered media. However, if the cover is absent then there are only three noticeable sub-pulses, which are caused by THz radiation multi-reflections from the left and right medium layer faces and interference pattern induced by these reflected signals. The first reflected sub-pulse in Fig 2A is usually named as the main sub-pulse and it occurs directly due to the THz pulse reflection from a medium layer face. Then, we observe much more sub-pulses in the reflected signal. They can usually be exploited with success for the substance detection and identification.

### 4.3 Fourier spectra

The Fig 2 demonstrates the comparison of the signal spectra, which are calculated as the absolute value of the Fourier transform using the well-known formula:

$$E_{refl\backslash tran}(\omega_{n})=\left|\sum_{k=1}^{N\prime }E(z_{refl\backslash tran},t_{k})\text{exp}(-\frac{2\pi it_{k}}{L}n)\right|,\;\omega_{n}=\frac{2\pi }{N\prime }n,\;t_{k}=\frac{L}{N\prime }k.$$ (105)

Here *L* is the time interval (in this case, *L* = *L*_*t*_, *N*′ = *N*_*t*_ = 10^4^) or the spatial computational domain length (*L* = *L*_*z*_, *N*′ = *N*_*z*_ = 3200). This transform is applied to the measured signals *E*_*refl*_, *E*_*tran*_ and similar Fourier transform is also applied to the incident pulse electric field strength distribution *E*(*z*, *t =* 0). Note that in physical experiments the frequency *ν = ω*/2*π* is usually measured, that is why in the figures below we use this ordinary frequency instead of the frequency *ω*.

**Fig 3:**
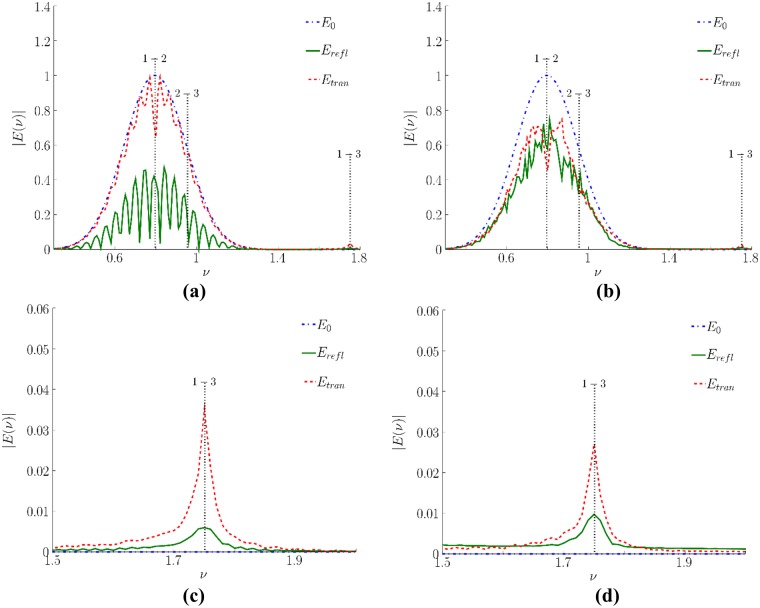
Comparison of the incident pulse spectra (*E*_*inc*_) with the spectra reflected from (solid line) or transmitted through (dashed line) the medium without cover (a) and spectra averaged over 32 random realizations for the dielectric permittivity in layers of a disordered structure (b). The spectra near the frequency, corresponding to 3–1 energy level transition, for uncovered and covered medium correspondingly (c, d) (dimensionless units).

As follows from the Fig 3A and 3B, the spectra are significantly disturbed because they possesses a strong modulation and it is even more powerful if a cover is absent. This is a consequence of the interference of different sub-pulses depicted in Fig 2. Obviously, the spectrum minima can be considered as the absorption frequencies and, thus, they may be treated as the false absorption frequencies [31]. To eliminate these mistakes, the sub-pulses should be analyzed separately.

At the same time, if covering of the medium is present, the THz pulse spectrum is not so much periodically modulated because the reflected signal is averaged over 32 random realizations. However, they exhibit irregular minima of various depth, which could be also interpreted as the absorption spectral lines of some substances while they are simply caused by the pulse multiple reflections from the faces of various layers and, consequently, an interference of appeared sub-pulses produces this spectrum modulation.

Other way for the substance identification may be achieved at using the substance emission frequency analysis. For the substance under consideration, a medium emission is present due to the energy level transition 3–1. We see that this frequency is absent in the incident pulse spectrum. Its appearance in the reflected pulse spectrum is due to the cascade mechanism of high energy level excitation of substance molecules. Because the spectrum shape near the frequency 1.75 (Fig 3C and 3D) is not distorted despite the presence of covering it is possible to use it for the substance identification.

**Fig 4:**
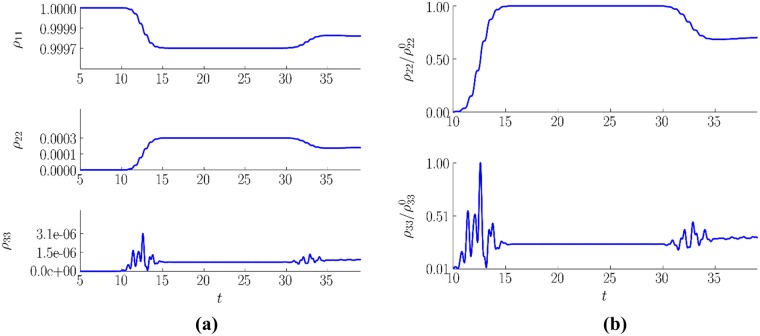
Time evolution of the energy level populations (a) in a section corresponding to the beginning of the medium z = 0. The detailed view of the initial stage for the second and third energy level populations normalized to their maximum values (b) (dimensionless units).

In Fig 4 the energy level population evolutions in the chosen section (*z* = 0) is shown. We stress that in our computer simulation, the parameters correspond to optically thin layer. Thus, the general behavior of the energy level populations in any medium sections is qualitatively similar to each of them. Hence, we depict a time evolution of the density matrix elements for a single section only. In these figures, it may be not evident that the third energy level is populated as a result of the 2–3 energy-level transition. Indeed, in Fig 4A, we see notable oscillations of the third energy level population and these oscillations are not synchronous to the second energy level population changing.

Let us notice that in [32] we have presented results corresponding to strong electric field strength amplitude (*E*_0_ = 2) at studying the transmitted signal only. In that case, the third energy level population increases after the second energy level population becomes high enough. At significantly lower electric field strength, it is necessary to use the correlation coefficient between a pair of the energy level populations as an estimation of the cascade mechanism presence for the high energy level excitation.

The correlation coefficient between two series of measurements containing *N*' numbers of data is given by the following formula:

$$corr(a,b)=\sum_{i=1}^{N\prime }\left(a_{i}-a_{mean})(b_{i}-b_{mean}\right)/\left(\sqrt{\sum_{i=1}^{N\prime }{\left(a_{i}-a_{mean}\right)}^{2}}\sqrt{\sum_{i=1}^{N\prime }{\left(b_{i}-b_{mean}\right)}^{2}}\right),$$ (106)

$$a_{mean}=\frac{1}{N\prime }\sum_{i=1}^{N\prime }a_{i},\;b_{mean}=\frac{1}{N\prime }\sum_{i=1}^{N\prime }b_{i},$$ (107)

where, *a*_*mean*_, *b*_*mean*_ are the mean values of the measured variables. This formula application to the energy level populations in the time interval [10, 35] with *N*' = 2500 yields the following correlation magnitudes:

$$corr(\rho_{11},\rho_{22})=-1,\;corr(\rho_{22},\rho_{33})=0.781,\;corr(\rho_{33},\rho_{11})=-0.71.$$ (108)

The correlation of the energy level populations with the instantaneous electric field strength in this section of the medium gives us the following values:

$$corr(\rho_{11},{E|}_{z=0})=0.001,\;corr(\rho_{33},{E|}_{z=0})=-0.14.$$ (109)

Thus, from Eq (106) it can be seen that the populations of the first and second energy levels are tightly connected and are uninfluenced by the instantaneous value of the electric field strength (107). On the other hand, the third level population is significantly correlated with the electromagnetic field due to non-resonant interaction. That means, when the electromagnetic pulse propagates in a medium, the electric field causes its polarization, related to non-diagonal elements of the density matrix *ρ*.

In accordance with invariants of Maxwell-Bloch equations (32), the appearance of the polarization induces temporary shifts in the energy level populations. In the case of three level medium, the invariant is written as follows:

$$\sum_{m=2}^{3}\sum_{n=1}^{m-1}{\left(\rho_{mm}(z,t)-\rho_{nn}(z,t)\right)}^{2}+6\sum_{m=2}^{3}\sum_{n=1}^{m-1}{\left|\rho_{mn}(z,t)\right|}^{2}=const(z).$$ (110)

As soon as external electric field strength decreases, the substance polarization decreases also, and consequently the populations return to their initial values. This is non-resonant process unrelated with the medium emission.

Nevertheless, the medium polarization is not only the process influencing the third energy level population evolution. High correlation between the second and third level *corr*(*ρ*22, *ρ*33) is more notable. That means that the second energy level population is significant factor governing the third energy level population evolution and the third energy level excitation takes place due to the cascade mechanism.

Thus, there are three processes that influence the population of the third level. One of them is the medium non-resonant polarization. Other two of them are resonant transitions between the second and third energy levels and between the third and first energy levels. The first process can cause the first growth of the third energy-level population *ρ*_33_ before of the second level population *ρ*_22_ growth. This process is not accompanied by electromagnetic field energy absorption and does not result in non-zero value of the third energy level population changes after the pulse action ending. Therefore, as soon as the field strength decreases, the population returns to the initial state without any medium emission. Other two processes are responsible the population relaxation process, which is several orders of magnitude slower than the energy level excitation processes.

### 4.4 Spectra of sub-pulses

In our previous papers [29], [30] we have shown that the detection and identification of a substance using the THz radiation measured in the reflection mode can be effective at analyzing the sub-pulses following the main pulse reflected from the substance face. In this case, we get information about the substance absorption frequencies. Below we provide a similar analysis for a medium covered by disordered structure. However, another our purpose is to observe also a substance emission at the frequencies corresponding to transitions from the high energy levels excited due to the cascade mechanism. To obtain the sub-pulse spectra, the Fourier-Gabor transform [48] is applied to the signal *E*(*Z*_*refl*\*tran*_, *t*_*k*_) in the corresponding time intervals.

It should be noted that if the signal amplitudes at the left and right ends of the time interval differ then the effect, which is known as the spectrum spreading [49], occurs. As a result, false frequencies appear in the signal spectrum. They can be suppressed by using the window weight function that tends to zero toward the ends of the time interval. For this purpose, at each time interval, which contains the corresponding sub-pulse, we use the Fourier-Gabor transform [48] with window function

$$G(t)=\text{exp}{\left(-\left((t-t_{I,II,III\ldots }\right)/\theta_{I,II,III\ldots }\right)}^{KG}),$$ (111)

which is a near-rectangular window. Therefore, we obtain:

$$E_{refl\backslash tran}^{I,II,III,\ldots }(\omega_{n})=|\sum_{k=1}^{N\prime }G(t_{k})\cdot E(z_{refl\backslash tran},t_{k})\cdot \text{exp}(-\frac{2\pi it_{k}}{L}n)|,\;\omega_{n}=\frac{2\pi }{N\prime }n,\;t_{k}=\frac{L}{N\prime }k,$$ (112)

where *E*_*I*,*II*,*III…*_(*ω*_*n*_) is the spectrum of the sub-pulse located in the corresponding time interval marked in Fig 2. Time *t*_*I*,*II*,*III…*_ denotes a centre the corresponding time interval and *θ*_*I*,*II*,*III…*_ denotes the window half-length. As before, *L* is the time interval, where the signal is analyzed, and *N*' is the number of time nodes in this time interval (*L* = *L*_*t*_, *N*' = 10^4^). The parameter *KG* in Eq (111) is even and characterizes the steepness of the window function at the ends of a time interval. In our simulation, this parameter was taken to be equal to 20 for the window function *G*(*t*) to fall off sharply.

**Fig 5:**
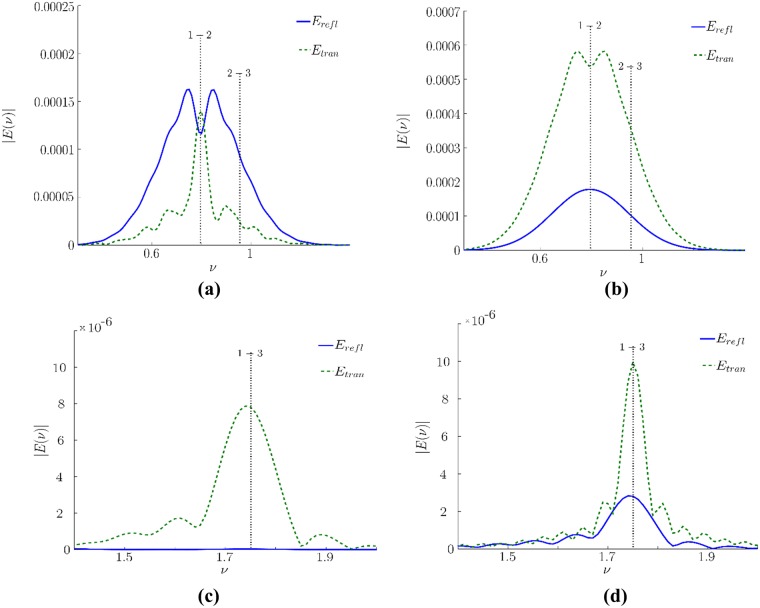
The first sub-pulse (area I in Fig 2) spectrum (a, c) and the second sub-pulse (area II in Fig 2) spectrum for the signal reflected from and transmitted through a medium without covering. The spectra (c, d) are shown in the higher frequency range (dimensionless units).

Fig 5 shows the sub-pulse spectra for the signals reflected from and transmitted through an uncovered medium. The transform window edges and the signal partitioning can be seen in the Fig 2A. In Fig 5A the spectrum modulation is absent at all. Apparently, the first reflected sub-pulse depicted in the Fig 5A is formed due to the reflection by the left face of the medium and thus, does not propagate inside the medium and, therefore, does not interact with it. This reflected pulse spectrum contains no information about the medium absorption frequencies, and the spectrum shape is the same as that for the incident pulse (Fig 3A).

The second sub-pulse (Fig 5B) appears due to multi-reflections from the right and left faces of the medium before being detected at the left section *E*_*refl*_. The absorption frequency corresponding to the transition 1–2 (the frequency 0.8) is noticeable in its spectrum as well as weak spectral intensity maximum at the frequency 1.75 corresponding to the cascade mechanism of high energy level excitation. The absorption frequency corresponding to the energy level transition 2–3 is not seen (Fig 5B) because of weak increasing of the second energy level population. As follows from Fig 4A, the latter remains quite low: thousand times less than the population of the first energy level. Therefore, the spectrum dip, corresponding to this energy level transition, is quite small.

In opposite, both transmitted sub-pulses exhibit notable spectrum peculiarities. The first one contains the absorption frequency 0.8. The second sub-pulse spectrum (Fig 5B and 5D) exhibits a substance emission at the frequencies 0.8 and 1.5. Hence, we observe the spectral maximum instead of spectral minimum at the frequency corresponding to the 1–2 energy level transition. The frequencies corresponding to the energy level transition 2–3 or 3–2 is not observable at all here. It should be stressed that in the spectrum of the second sub-pulse transmitted through a medium, some additional false absorption frequencies occur.

**Fig 6:**
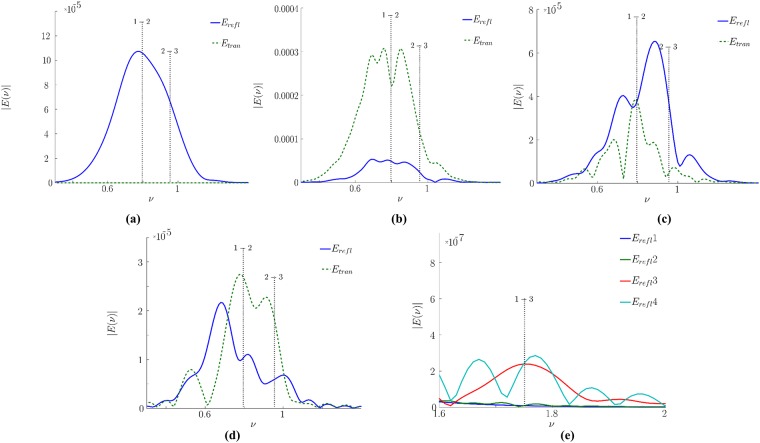
Spectrum of sub-pulses reflected from and transmitted through the covered medium and belonging to the time interval I (a), II (b), III (c), IV (d) in Fig 2B. The reflected sub-pulse spectra in the vicinity of the frequency 1.75 (emission frequency) corresponding to the energy level transition 3–1 (e).

Let us discuss the corresponding spectra of the sub-pulses reflected from or transmitted through the medium covered by disordered structure. The temporal partitioning of the sub-pulses can be seen in the Fig 2B. In Fig 6 the corresponding sub-pulse spectra are depicted. We see that the main reflected pulse spectrum in Fig 6A is slightly different from the incident pulse spectrum.

The main transmitted pulse, located in the time region II (Fig 2B), exhibits the absorption frequency 0.8 presence in the spectrum (Fig 6B). Nevertheless, the pulse spectrum contains the false absorption frequency, which is equal to 0.7. The second reflected sub-pulse spectrum (Fig 6B) contains only the false absorption frequencies, which do not belong to a medium, and they are a result of superposition of the pulses caused by electromagnetic field multi-reflections from the layered structure.

In the time area III (Fig 2B), the second transmitted and the third reflected pulses are located (Fig 6C). The electric field strengths of these pulses are 10 times less than the ones for previous sub-pulses. Nevertheless, the emission at the frequency 0.8 related to 2–1 energy level transition can be observed in the transmitted sub-pulse spectrum. At the same time, the absorption frequency corresponding to 2–3 energy level transition is noticed together with several absorption frequencies. We stress that the third reflected sub-pulse is the first reflected one that contains the absorption frequencies related to the medium. However, it contains also the false frequencies about of 0.5 and 0.6 dimensionless units, and there is a spectrum minimum at the frequency 1.01, which is shifted away from the 2–3 transition resonance frequency.

The spectrum of the third transmitted sub-pulse, located in time interval IV, contains maximum at the frequency about of 0.8 (this means a substance emission presence (Fig 6D)) and it contains also a number of false absorption frequencies. The fourth reflected sub-pulse is distorted as well. In the frequency range *ν*<0.7 the spectrum shape is similar to that of the third reflected sub-pulse. Thus, the same mechanism of the spectrum distortions exists. However, the other part of the spectrum is quite different: the spectrum minimum at the frequency 0.8 is still present but it is shifted to the range of lower frequencies, and the spectrum minimum at the frequency 0.9 is observed, unlike the third sub-pulse in which the spectrum minimum is located at the frequency 1.01.

Fig 6E demonstrates comparison of reflected sub-pulses spectra near the frequency 1.75 corresponding to 3–1 energy level transition. For the first and second sub-pulse, the spectral intensity maximum at this frequency is absent. The third and fourth sub-pulse spectra have the maximum near the frequency 1.75. It means that those sub-pulses can be used for the substance identification. However, the fourth sub-pulse is very weak and is significantly distorted even in this frequency range.

Thus, we have demonstrated the cascade mechanism of high energy level excitation and the emission frequency observation corresponding to the transition 3–1 in the spectra of both pulses reflected from or transmitted through the substance. Since this frequency is observed in the case of the covered medium, it may be used with success for the detection and identification of the substance.

## 5. Conclusions

We develop the conservative finite-difference scheme for the problem describing an interaction of the THz pulse, containing a few cycles, with the multilevel medium in the framework of the Maxwell-Bloch equations. This finite-difference scheme is based on the Crank-Nicolson scheme with using a new approximation of the artificial boundary conditions on the basis of the Cabaret scheme. For a solution of the corresponding nonlinear difference equations, the iterative process is proposed and its convergence condition is written.

We generalize the Bloch invariant for a multilevel medium and the conservativeness of the developed finite-difference scheme with respect to this invariant as well as to the invariants of the Maxwell’s equations were proved. The proposed finite-difference scheme allows to control the positiveness property of the energy level populations at computer simulation by choosing the mesh step in appropriate way.

We have demonstrated that the disordered structure covering of a medium results in appearing of the false absorption frequencies. This effect arises from complicated interference of the multiple sub-pulses reflected from various faces between layers of the disordered structure.

We showed the appearing of the emission frequencies belonging to the range of high frequencies in the sub-pulses spectra due to the cascade mechanism of high energy level excitation for both transmitted and reflected THz signals. The disordered cover less distorts the spectral intensities of these frequencies. Therefore, they can be used with success for the substance detection and identification.
